# Medical Student Involvement in a Human Rights Program: Impact on Student Development and Career Vision

**DOI:** 10.5334/aogh.2940

**Published:** 2020-10-08

**Authors:** Stephanie M. Schonholz, Madison C. Edens, Axel Yannick Epié, Sophie Karwoska Kligler, Kim A. Baranowski, Elizabeth K. Singer

**Affiliations:** 1 Icahn School of Medicine at Mount Sinai, New York, US; 2 Mount Sinai Human Rights Program, Icahn School of Medicine at Mount Sinai, New York, US; 3 Emergency Medicine and Medical Education, Icahn School of Medicine at Mount Sinai, New York, US; 4 Global Health Division, Department of Emergency Medicine, Mount Sinai St. New York, US

## Abstract

**Background:**

There is consensus among many medical school deans that exposure to human rights is a necessary component of physician training [7,8], however little is known about the impact of engagement in human rights programs on students’ personal and professional development [15,16,17,18,19,20,21,22,23,24,25,26,27,28].

**Objective:**

This study aimed to examine medical students’ experiences in the Mount Sinai Human Rights Program (MSHRP), their motivations for involvement, and the possible influence of engagement on their professional identities, personal growth, and career choices.

**Methods:**

Through semi-structured interviews, this qualitative study gathered the experiences of 15 fourth year and recently graduated medical students who participated in the comprehensive training, research, and direct service opportunities provided by the program. Responses were coded using a content analysis approach.

**Findings:**

The results of this research highlight the motivations behind students’ involvement in a medical human rights program, as well as the challenges they experienced engaging with this work. The study captured students’ perceptions of the role of the program on their personal growth, clinical skills, and career vision. Nearly all the students interviewed indicated they developed important, clinically applicable skills that enhanced their traditional medical education. Students indicated that their participation directly influenced their professional identities and future career directions by reinforcing previous interests in human rights and social justice work, impacting medical specialty and residency program selections and fostering commitment to working with immigrant populations.

**Conclusions:**

The results of this study indicate that longitudinal involvement with the MSHRP contributed to the acquisition of important clinical skills that were not otherwise attained in students’ early medical education. Findings suggest that there is significant opportunity for clinical and leadership development outside the traditional preclinical and clinical setting, and that exposure to human rights education shapes students’ professional identities and career paths. Finally, the findings highlight the essential role of human rights and social justice in medical education.

## Introduction

We are currently in the midst of the largest humanitarian crisis since World War II. In 2018, over 70 million people were forcibly displaced from their homes due to persecution, conflict, violence, or human rights violations. Asylum seekers represent 3.5 million individuals worldwide [1]. An asylum seeker is a person who is seeking protection from persecution from inside a receiving country or at a port of entry [2]. As of December 2019, there were over one million pending asylum applications which have overwhelmed the U.S. immigration system [3].

Healthcare providers often treat immigrants, many of whom may be refugees and asylum seekers. Studies have demonstrated that although physicians who work in urban areas with large immigrant populations will certainly care for survivors of torture, persecution, or ill treatment in their daily practice, this population often remains invisible [4,5,6]. In aiming to better identify and treat these survivors, there has been an increased focus on incorporating immigrant health and human rights curriculum into medical education [7]. Yet in a survey of U.S. medical schools, although 76% of deans reported that they believe human rights knowledge is important to medical education, only 37% of those same programs incorporated health and human rights into their curricula [8], indicating that additional work is needed to expand such training. Outside of formal classroom learning and in response to the current global refugee crisis, many U.S. medical schools have supported the development of student-led human rights medical clinics and programs that serve asylum seekers. In fact, medical student groups that focus on human rights and forensic evaluations exist in more than 19 medical schools across the U.S. [9]. A recent study demonstrated that student-run clinics have performed more than 1,600 forensic evaluations for asylum seekers, with students often scheduling assessments, serving as scribes during evaluations, and aiding in connecting asylum seekers to continuity medical care and social services [10]. Detailed descriptions of programs’ structures, management, and participant roles have been well documented [11,12,13,14].

Although such programs and human rights education are thought to enable students to drive meaningful change in their communities, research exploring their direct impact on students is limited [15,16]. One prior study explored medical students’ work with asylum seekers and refugees, as a way to increase cultural competency and as a teaching modality designed to reach beyond the traditional classroom format of lectures and discussions [17]. Other studies have investigated the influence that clinical experiences with asylum seekers and refugees have had on medical learners, but have not directly interviewed these students [18,19,20]. A further literature review of medical student experiences in human rights work produced one student reflection [21], a study that focused on the development and evaluation of a medical school curriculum [22], a longitudinal assessment of an international global health student elective [23], and a survey evaluating student participation in a refugee resettlement program [24]. Still, other studies have examined the effect that participating in a human rights program and working with asylum seekers have on residents and physicians [25,26,27,28].

While the benefits of human rights education have been acknowledged, it is also recognized that little data exists on the perspectives of medical students involved in these programs and the perceived impact they may have on students. To our knowledge, there are no in-depth, empirical studies examining the ways in which working with asylum seekers and participating in a human rights program directly impact medical students personally or professionally. There is, thus, a need for studies to demonstrate the impact of such education on students’ knowledge base, skill set, and career visions.

The Mount Sinai Human Rights Program (MSHRP) is a student-run, faculty-directed organization at the Icahn School of Medicine at Mount Sinai (ISMMS) that serves asylum seekers to the U.S. Founded in 2013, the program has expanded greatly and currently serves nearly 200 clients a year from over 60 countries. The program provides asylum seekers with *pro bono* forensic evaluations that document the sequelae of human rights violations, as well as access to continuity healthcare and social services. Through involvement with the MSHRP, students are exposed to the logistics of program management, opportunities to conduct research, and experiences with training the community about health and human rights. For students in the clinical years, the program reinforces interviewing, diagnosis, and cultural competency skills. In 2019, the inaugural class of MSHRP students entered their final year at ISMMS or became graduates. This study was designed to gather students’ experiences in the program, their motivations for involvement, and the possible influence of engagement on their development, including their professional identities, personal growth, and career choices.

## Method

### Participants

Participants included 15 current and former ISMMS medical students who actively participated in the MSHRP, beginning in the first year of medical school. The majority of participants were current fourth year medical students (*n* = 13, 87%) while a smaller number were medical students who had graduated one to two months prior to the interviews and were now in post-graduate training (*n* = 2, 13%). Participants identified as men (*n* = 3, 20%) and women (*n* = 12, 80%) and ranged from 24 to 29 years of age (*M* = 26.3). The participants identified as Black (*n* = 1, 7%), Asian (*n* = 2, 13%), Hispanic (*n* = 1, 7%), and White (*n* = 7, 47%). Three participants (20%) identified as bi-racial, including two (13%) as White and Hispanic and one (7%) as Black and Pacific Islander. Most (*n* = 13, 87%) reported prior experience in human rights work, including extracurricular activities, professional experience, and undergraduate coursework.

### Procedure

Eligible participants were: 1) active participants in the MSHRP, and 2) enrolled in the fourth year at ISMMS or a graduate of the previous academic year. We recruited participants through email announcements, which included a study description and contact for the principal investigators; 88% of the students contacted (*n* = 17) agreed to participate. The Institutional Review Board approved the study and all participants provided informed consent.

### Measures

Participants completed a brief demographic questionnaire and semi-structured interview protocol (**Appendix**). We conducted interviews by telephone which were audio recorded, transcribed, and de-identified by the research team members before analysis.

### Data Analysis

We analyzed the resulting data using the following content analysis steps: (a) design, (b) unitizing, (c) sampling, (d) coding, (e) drawing inferences, and (f) validation [29]. After determining the purpose of the study and identifying the participants’ responses as the unit of analysis, we sampled medical students formally or currently engaged in the MSHRP. Next, we independently created codes and organized the interview data into themes. We met and argued codes to consensus to reduce bias. By analyzing the themes that emerged across participants, we developed inferences on the impact of the program on students’ personal and professional growth. Lastly, we validated our findings by ensuring a “balance between participant meaning and researcher interpretation [30].” We used an auditor with experience in qualitative research to ensure coding accuracy, incorporated direct participant quotes to support the themes, and elicited feedback from participants to increase the validity of the findings.

## Results

Through our systematic analysis of the interviews, we distilled study participants’ experiences into six themes: motivation for involvement, clinical application and skills, personal impact and growth, challenges, career vision, and human rights and social justice in medical education. The resulting themes and categories are reported with their accompanying frequencies (Table 1).

**Table 1:** Medical students’ experiences with the MSHRP (*n* = 15).

Theme	Category	No. of cases (%)

Motivation for involvement		
	Passion for service, social justice, immigration work	11 (73%)
	Faculty leaders	6 (40%)
	Student members	5 (33%)
	Familial experience with immigration	2 (13%)
Clinical application and skills		
	Navigating the medical-legal process of asylum and affidavit writing	15 (100%)
	Trauma-informed clinical skills	11 (73%)
	Leadership and management	9 (60%)
	Program establishment, development, and growth	8 (53%)
	Establishing partnerships and identifying social services	6 (40%)
	Conducting and presenting research	3 (20%)
Personal impact and growth		
	Provided a formative medical school experience	13 (87%)
	Provided a community of like-minded peers and mentors	8 (53%)
	Inspired by asylum seekers	5 (33%)
	Increased awareness of current migrant crisis	4 (27%)
Challenges		
	Program operations	9 (60%)
	Time management	9 (60%)
	Establishing roles and responsibilities	6 (40%)
	External barriers	6 (40%)
	Emotional strain	2 (13%)
Career Vision		
	Motivated to pursue social justice and human rights work	15 (100%)
	Inspired to work within medical-legal systems	11 (73%)
	Influenced choice of residency program and/or medical specialty	10 (67%)
Human rights and social justice in medical education		
	Provides social context of patient care	10 (67%)
	Fundamental to medicine	8 (53%)
	Strengthened by a dedicated and longitudinal curriculum	7 (47%)
	Offers diverse opportunities for hands on experience	6 (40%)
	Requires institutional and faculty support	4 (27%)

Abbreviation: Mount Sinai Human Rights Program (MSHRP).

### Motivation for Involvement

Participants reported both internal and external motivating factors for joining the program. They described having a passion for service, social justice, and immigration work (*n* = 11, 73%). Others highlighted the desire to collaborate with the faculty leaders (*n* = 6, 40%) and student members (*n* = 5, 33%). Two participants (13%) mentioned familial experience with immigration as a motivation, as exemplified by:

“My family is from Honduras, so I think given our current political situation, I started becoming really involved in the conversation of unaccompanied minors in college. But I’d always known Central American politics and the things people were escaping growing up. It was so valuable to be able to put that knowledge and to make use of this itch that I had to really give back…”

### Clinical Application and Skills

All the participants (*n* = 15, 100%) reported developing important clinical skills through their participation in the MSHRP. Many (*n* = 9, 60%) reported increased leadership and management abilities, including the capacity to “manage a team, manage tasks across multiple channels, or corral and motivate people [to work towards] a much larger vision.” Over half of the students (*n* = 8, 53%) noted that they learned to establish, develop, and grow a program, including “building something from the ground up” while “deciding how we wanted it look and how we wanted it to run.”

Participants especially appreciated the clinical application of their experiences during their pre-clinical years and noted the usefulness of these skills during their subsequent clinical rotations. Over two-thirds of students (*n* = 11, 73%) reported developing trauma-informed clinical skills, such as expressing empathy, careful physical exams, in-depth psychiatric evaluations, and sensitive and efficient interviewing. One participant elaborated, “You learn things in such detail in this setting. It’s helped me as I’ve progressed through medical school to approach every patient I encounter with a fine-tuned comb, with this in-depth lens.” Students also indicated they learned to establish partnerships and identify social services (*n* = 6, 40%), by collaborating with lawyers to connect asylum seekers to support for housing, food assistance, transportation, education, and employment.

All participants (*n* = 15, 100%) indicated gaining foundational knowledge in navigating the medical-legal process of asylum and affidavit writing. One noted, “I learned what it takes to witness someone’s journey and effectively document medical and psychiatric sequelae [of persecution].” They also reported that drafting affidavits for forensic settings led to skills in translating medical jargon into layperson’s terms. Finally, a few participants (*n* = 3, 20%) reported that faculty mentorship helped them gain confidence in conducting, interpreting, and presenting research using program data.

### Personal Impact and Growth

Nearly all the participants (*n* = 13, 87%) referred to the MSHRP as a formative medical school experience, describing the work as “unique,” “rewarding,” “worthwhile,” and, for some, the “most important medical school experience.” Several commented that they appreciated the opportunity to address issues they were passionate about, with one participant explaining, “Especially in the pre-clinical years, it’s really easy to lose sight of why we’re studying so much … being involved helped remind me of the things that I care about.” Others highlighted the value of their contributions, as exemplified by:

“When [an asylum seeker] I helped write an affidavit for would end up getting a positive outcome in their court case, that really spoke to the impact we can do. So, it taught me that, yeah…even though we don’t have an MD, we can really help change people’s lives.”

Some participants (*n* = 4, 27%) also reported that their work with the MSHRP facilitated an increased awareness of the current migrant crisis. They stated that they relished having a platform to respond to current events that were “out of our control” and “to actually be involved in these discussions.” Several (*n* = 5, 33%) commented that they were inspired by asylum seekers they served. One participant remarked, “It’s been powerful, humbling, really motivating for pushing through medical school and thinking about what I want to do with this incredible privilege of a degree.” About half of the sample (*n* = 8, 53%) commented that collaborating with a community of like-minded peers and professionals fostered mentorship and an appreciation for their colleagues’ contributions and dedication to the program.

### Challenges

The challenges participants encountered in the program ranged from personal to systems-based. A little over one-third (*n* = 6, 40%) stated that they were met with external barriers including financial, institutional, and political obstacles. One participant remarked, “It’s challenging to know that that money will run out and that we’re constantly having to pour our time and energy and resources into fundraising … when that time and energy, and those resources, could be poured into [helping] asylum seekers.”

A few students expressed disillusionment, largely due to current immigration policies and the asylum application process itself. One participant stressed:

“I think the amount of effort that I learned it takes for someone who doesn’t speak the language, to somehow immigrate to this country, find a lawyer who might speak their language or find a translator who does, to then be able to articulate their story, and apply for asylum. To get through that process, somehow still surviving as a human in the world, and then their lawyer has to contact a physician for a formal affidavit, and *that* is how they get asylum? That, I think, was just really astounding.”

Nearly two-thirds of participants (*n* = 9, 60%) highlighted challenges in program operations. They noted difficulties associated with rapid growth in program membership, varying levels of student commitment, and the transition of leadership inherent in any student-led program. Students also referenced difficulties associated with time management (*n* = 9, 60%). They recounted the challenge of balancing coursework, exam preparation, and clinical responsibilities with their intended work with the MSHRP. Despite this strain, participants expressed regret that they were unable to contribute more of their time in medical school to the program. One student noted, “I always felt like this is what I’m actually interested in. This is what makes me really happy, but I don’t feel like as much of my time as I would have liked is going here.”

While many participants cited the student-faculty hybrid organizational model as a strength of the program, some (*n* = 6, 40%) also described the challenges related to establishing roles and responsibilities between faculty and students:

“There’s always been a little blurry line between the faculty responsibilities and the student responsibilities, and I think in any given year that line shifts depending on how much student leadership wants to take on and what the questions of the moment are.”

They also commented on tensions associated with dividing responsibilities between students, who have a range of interests and degrees of involvement in the program:

“Getting the organization off the ground and having a clear division of tasks was hard. When a lot of people want to feel ownership and contribute, it can be hard to focus on what actually needs to be done.”

A minority of participants (*n* = 2, 13%) cited the emotional strain of working with asylum seekers. They commented that they experience guilt, “Feeling like you didn’t do enough, and that you could have been the reason that things could have been better, and now they’re not, you know?” They also discussed the cumulative emotional toll of helping document evidence of physical, sexual, and psychological violence.

### Career Vision

Despite the challenges students encountered in their program involvement, they reported that it profoundly affected their visions for their future careers. Every participant (*n* = 15, 100%) reported motivation to pursue social justice and human rights work as a result of their engagement in the MSHRP. One participant commented, “I anticipate that I will be involved in this kind of work for the rest of my career … And that’s about as big a development in professional identity that you can get through an extracurricular.”

Many students remarked that they did not realize a career in medicine that focuses primarily on human rights and social justice was an option. Participants attributed these expanded perspectives to the program’s faculty leaders and evaluators, who served as role models for their emerging professional identity:

“It’s been one of the foremost experiences that’s shaped my medical experience from a mentorship perspective, and reframing maybe what I want to do in the future and how I consider what a career in medicine could look like.”

Over two-thirds of the group (*n* = 11, 73%) stated that they were now inspired to work within medical-legal systems, to use their medical degree to support and advocate for initiatives they care about. There was a resounding plan to conduct forensic evaluations and write affidavits on behalf of asylum seekers once licensed. Some students also stated intentions to help shape immigration and asylum policy or pursue a career in politics.

Two-thirds of participants (*n* = 10, 67%) reported that their engagement in the MSHRP influenced their choice of residency program and/or medical specialty. One participant asserted, “I know that I want to be at a [residency] with that same outlook, attitude, and willingness to support people who have suffered from human rights abuses.” A subset of participants even commented that their participation with the program influenced the area of medicine they decided to pursue: “I knew I wanted to work with underserved populations, but I think [the program] solidified what I wanted to do.” Participants currently in residency reported having actively sought out and joined similar organizations at their new institutions.

### Human Rights and Social Justice in Medical Education

More than half of the students (*n* = 8, 53%) asserted that human rights and social justice are fundamental to medicine and to the education and training of any physician. They remarked that physicians have a “moral responsibility” to utilize social justice to better understand and empower their patients. One participant elaborated, “Social justice and human rights should be at the center of medical education. I think it’s one of the most helpful, inspiring, and useful frameworks to give context as to what responsibilities doctors have, and the idea that medicine … does not exist in a vacuum.”

Two-thirds of participants (*n* = 10, 67%) described how this type of curriculum provides a social context to patient care. They stated that understanding the social, political, and immigration systems impacting their patients and communities is essential to increasing wellbeing. They suggested that clinicians consider their clients’ distress from a holistic perspective: “Someone could have a broken leg because they fell off their bike, but also have just entered this country and be suffering from the stressors of being undocumented or being an asylum-seeker.”

Additionally, participants made recommendations for strengthening human rights and social justice in medical education. Approximately half (*n* = 7, 54%) asserted that medical education would be strengthened by a dedicated and longitudinal curriculum that increases students’ exposure to these issues and their ability to participate in conversations about human rights across specialties within the hospital system. They also encouraged medical schools to teach trauma-informed care as part of the physical exam and highlight the sequelae of female genital mutilation/cutting during courses on sexual health and reproduction. In addition, nearly half the group (*n* = 6, 40%) noted that human rights programs offer diverse opportunities for hands-on experience. Finally, participants advised that a program like the MSHRP can only succeed with the support of faculty and the department of medical education (*n* = 4, 27%), and stressed that strong faculty leadership “drives the ship forward” and provides necessary program continuity.

## Discussion

This study uses the voices of students to describe the impact of their participation in a human rights program. The students in our sample were motivated to participate because of their passion for service, social justice, and immigration work. In addition, students reported gaining the knowledge needed to establish and develop a program, and increasing their trauma-informed clinical skills through hands-on opportunities. These findings echo the diverse ways in which human rights education may be acquired and conceived, and through which health professionals may come to view the relevance of human rights norms for their work [31]. Furthermore, almost all of the students viewed participating in the program as a powerful and formative experience during medical school, which contributed to an increased awareness of current events surrounding the migrant crisis, led to personal growth, and inspired new ways in which to use their expanding expertise. Therefore, this platform for education in human rights is one which may empower students to act on behalf of vulnerable patients and to advocate for social justice.

The findings also highlighted how involvement with a human rights program shaped students’ professional identities and visions for their careers in a way that prior investigations had not explored. Students unanimously reported that their involvement with the MSHRP motivated them to pursue a career in social justice, policy, or human rights and to use their medical degrees to support initiatives about which they were passionate. The majority also sought out post-graduate residencies and specialties in which they could build on the skills they had acquired and continue to work with underserved populations. Thus, integrating human rights programs into medical schools may ultimately increase the ability of the healthcare system to respond to the needs of asylum seekers, immigrants, and other underserved patients.

This study also set out to explore the role and relevance of human rights education in shaping physicians and their understanding of patient care. Students viewed the human rights and social justice framework as crucial to their development as professionals. They felt that it gave them a context in which to better understand their own responsibilities to their patients, as well as the larger social setting and political system in which medicine is practiced. These observations have been reflected by others who have considered the role of human rights in patient care and have used a human rights lens to examine systemic issues in health [32].

A few challenges, both on the individual and system-level, were also noted by participants, and underscored some of the ways in which student-led, faculty-directed human rights programs may be improved. Students noted external barriers, specifically institutional obstacles and the financial pressure to fundraise, which detracted from time spent on direct service for asylum seekers. As with many not-for-profit endeavors, human rights programs often devote significant time and resources to secure necessary funding. Broader support for human rights programs from within large academic institutions may shift some focus back to patient care. Furthermore, students noted strain in program operations at times of rapid growth in membership, secondary to varying levels of student commitment and during the annual transition of student leadership, where challenges to maintaining smooth operations surfaced. The unique student-faculty hybrid nature of the MSHRP’s leadership was also cited by students as a factor which made the clear delineation of roles and responsibilities more challenging at times. Yet, the successes of the MSHRP in its ability to serve an increasing number of clients each year and to educate an expanding segment of the medical school and community [33] demonstrates that success is achievable, a fact that may encourage students at other institutions who are seeking opportunities to start a human rights program.

Finally, results highlight the cumulative emotional toll and exposure to secondary trauma related to service provision with survivors of torture. The risk of vicarious trauma in human rights advocates is well documented in the literature [34,35] and the MSHRP responds to the need for trauma-informed organizational development by facilitating wellness sessions. During these voluntary meetings, licensed mental health professionals provide psychoeducation on the prevention of burnout and secondary traumatic stress, as well as support the development of compassion satisfaction and vicarious resilience.

The findings suggest that human rights and social justice can be strengthened in medical education through a longitudinal and dedicated curriculum, beginning in the pre-clinical years, that would increase exposure to these issues throughout the institution and hospital system. Participants also noted that human rights programs, such as the MSHRP, require the consistent support of the medical school, as well as identified faculty champions, the latter providing an overall vision for the program and continuity.

## Conclusion

In this study, we examined fourth year medical students’ and recent graduates’ experiences in a human rights program, their motivations for involvement, and how their engagement influenced their professional identities, personal growth, and career choices. After conducting and analyzing 15 semi-structured interviews using a content analysis approach, we found that involvement in the human rights program aided in medical students’ personal and professional development as it provided a forum for experiences outside of traditional academic and clinical medical training. Given the paucity of in-depth, empirical research examining medical students’ experiences in programs such as the MSHRP, our study offers a novel perspective of the significant impact human rights education may have on future doctors’ career choices and personal growth. Future qualitative studies involving larger cohorts across medical education institutions will be important in order to further assess the direct impact of human rights programs on medical students. Such studies will also provide a broader understanding of our findings and the role human rights programs play in medical education.

## Limitations

Potential study limitations included issues related to sampling, as all participants originated from one human rights program under the umbrella of a single institution. As such, the generalizability to a wider community of medical students and student-led human rights programs in other geographic locations is limited. Future studies should investigate the experiences of a broader base of students involved in human rights programs across the nation. Finally, this qualitative study did not include a comparison cohort of students who were not MSHRP participants. Ongoing surveys of MSHRP current and graduated students will be undertaken to better understand how they integrate health and human rights into their careers and remain engaged in these issues throughout their professional development.

## Appendix

### Interview Protocol

1. How did you come be involved with the MSHRP?
2. To what extent did you consider yourself active in social justice and/or human rights work before joining the MSHRP?
3. Describe the skills you developed through your work with the program.
4. How has your participation in the MSHRP positively impacted you?
5. What have been challenges associated with your participation in the MSHRP?
6. How has your participation in the MSHRP influenced your understanding of the role of social justice work in clinical medical education?
7. How has your involvement in the MSHRP shaped your professional identity?
8. In what ways, if any, has your involvement in the MSHRP influenced your future career goals?
9. What recommendations do you have to improve human rights education in medical training?
10. Is there anything else you would like to add about your experience participating in the MSHRP?
11. What was it like for you to participate in this interview?

## References

[1] UNHCR. Global Trends—Forced Displacement in 2018. https://www.unhcr.org/globaltrends2018/ (Accessed December 18, 2019).

[2] National Immigration Forum. Fact Sheet: U.S. Asylum Process 2019. https://immigrationforum.org/article/fact-sheet-u-s-asylum-process/ (Accessed December 18, 2019).

[3] TRAC Immigration. Immigration Court Backlog Tool: Pending Cases and Length of Wait in Immigration Courts 2020. https://trac.syr.edu/phptools/immigration/court_backlog/ (Accessed December 18, 2019).

[4] Eisenman DP, Keller AS, Kim G. Survivors of torture in a general medical setting: How often have patients been tortured, and how often is it missed? West J Med. 2000; 172(5): 301–304. DOI: 10.1136/ewjm.172.5.301 10832420 PMC1070871

[5] Crosby SS, Norredam M, Paasche-Orlow MK, Piwowarczyk L, Heeren T, Grodin MA. Prevalence of torture survivors among foreign-born patients presenting to an urban ambulatory care practice. J Gen Intern Med. 2006; 21(7): 764–768. DOI: 10.1111/j.1525-1497.2006.00488.x 16808779 PMC1924712

[6] Hexom B, Fernando D, Manini AF, Beattie LK. Survivors of torture: Prevalence in an urban emergency department. Acad Emerg Med. 2012; 19(10): 1158–1165. DOI: 10.1111/j.1553-2712.2012.01449.x 23009130

[7] Fitchett JR, Ferran E, Footer K, Ahmed N. Health and human rights: An area of neglect in the core curriculum? J Med Ethics. 2011; 37(4): 258–260. DOI: 10.1136/jme.2010.037556 21335572

[8] Cotter LE, Chevrier J, El-Nachef WN, et al. Health and human rights education in U.S. schools of medicine and public health: Current status and future challenges. PLOS ONE. 2009; 4(3): e4916 DOI: 10.1371/journal.pone.0004916 19293936 PMC2654657

[9] Ferdowsian H, McKenzie K, Zeidan A. Asylum medicine: Standard and best practices. Health Hum Rights. 2019; 21(1): 215–225. PMC6586957 31239628

[10] Sharp MB, Milewski AR, Lamneck C, McKenzie K. Evaluating the impact of student-run asylum clinics in the US from 2016–2018. Health Hum Rights. 2019; 21(2): 309–323. 31885459 PMC6927377

[11] Praschan N, Mishori R, Stukel N. A student-run asylum clinic to promote human rights education and the assessment and care of asylum seekers. J Stud-Run Clin. 2016; 2(2). https://studentrunfreeclinics.org/journalsrc.org/index.php/jsrc/article/view/18 (Accessed December 11, 2019).

[12] Chelidze K, Sirotin N, Fabiszak M, et al. Documenting torture sequelae: The Weill Cornell model for forensic evaluation, capacity building, and medical education In: Adjudicating Refugee and Asylum Status: The Role of Witness, Expertise, and Testimony. Online: Cambridge University Press; 2014: 1–248. DOI: 10.1017/CBO9781107706460.011

[13] Human Rights Initiative at the University at Buffalo. The value of medical students in support of asylum seekers in the United States. Fam Syst Health J Collab Fam Healthc. 2018; 36(2): 230–232. DOI: 10.1037/fsh0000326 29902039

[14] Francis ER, Goodsmith N, Michelow M, et al. The global health curriculum of Weill Cornell Medical College: How one school developed a global health program. Acad Med. 2012; 87(9): 1296–1302. DOI: 10.1097/ACM.0b013e3182628edb 22929431 PMC8052981

[15] Erdman JN. Human rights education in patient care. Public Health Rev. 2017; 38: 14 DOI: 10.1186/s40985-017-0061-8 29450086 PMC5809882

[16] Rowthorn V. Global/Local: What does it mean for global health educators and how do we do it? Ann Glob Health. 2015; 81(5): 593–601. DOI: 10.1016/j.aogh.2015.12.001 27036715

[17] Deliz JR, Fears FF, Jones KE, Tobat J, Char D, Ross WR. Cultural competency interventions during medical school: A scoping review and narrative synthesis. J Gen Intern Med; 11 2019 DOI: 10.1007/s11606-019-05417-5 PMC7018865 31705475

[18] Albritton TA, Wagner PJ. Linking cultural competency and community service: A partnership between students, faculty, and the community. Acad Med. 2002; 77(7): 738–739. DOI: 10.1097/00001888-200207000-00024 12114156

[19] Griswold K, Kernan JB, Servoss TJ, Saad FG, Wagner CM, Zayas LE. Refugees and medical student training: Results of a programme in primary care. Med Educ. 2006; 40(7): 697–703. DOI: 10.1111/j.1365-2929.2006.02514.x 16836544

[20] Stone H, Choi R, Aagaard E, et al. Refugee health elective. MedEdPORTAL. 2013; 9 DOI: 10.15766/mep_2374-8265.9457

[21] Ruchman SG. Asylum seekers: Our patients at the border. J Gen Intern Med. 2019; 34(12): 2908–2909. DOI: 10.1007/s11606-019-05218-w 31346907 PMC6854198

[22] Asgary R, Saenger P, Jophlin L, Burnett DC. Domestic global health: A curriculum teaching medical students to evaluate refugee asylum seekers and torture survivors. Teach Learn Med. 2013; 25(4): 348–357. DOI: 10.1080/10401334.2013.827980 24112205

[23] Imperato PJ. A third world international health elective for U.S. medical students: The 25-year experience of the State University of New York, Downstate Medical Center. J Community Health N Y. 2004; 29(5): 337–373. DOI: 10.1023/B:JOHE.0000038652.65641.0d 15471419

[24] Bernhardt LJ, Lin S, Swegman C, et al. The refugee health partnership: A longitudinal experiential medical student curriculum in refugee/asylee health. Acad Med. 2019; 94(4): 544–549. DOI: 10.1097/ACM.0000000000002566 30570498

[25] Mishori R, Mujawar I, Ravi N. Self-reported vicarious trauma in asylum evaluators: A preliminary survey. J Immigr Minor Health. 2014; 16(6): 1232–1237. DOI: 10.1007/s10903-013-9958-6 24306284

[26] Metalios EE, Asgary RG, Cooperman N, Smith CL, Du E, Sacajiu G. Teaching residents to work with torture survivors: Experiences from the Bronx Human Rights Clinic. J Gen Intern Med. 2008; 23(7): 1038–1042. DOI: 10.1007/s11606-008-0592-2 18612740 PMC2517941

[27] Baranowski KA, Moses MH, Sundri J. Supporting asylum seekers: Clinician experiences of documenting human rights violations through forensic psychological evaluation. J Trauma Stress. 2018; 31(3): 391–400. DOI: 10.1002/jts.22288 29786891

[28] Patel NA, Sreshta N, Frank A, Marlin RP, Boyd JW. Psychiatric resident participation in an asylum clinic: A single-institutional experience. Acad Psychiatry. 2019; 43(1): 56–60. DOI: 10.1007/s40596-018-0925-3 29687306

[29] Krippendorff K. Content analysis In: International Encyclopedia of Communication Volume 1 NY: Oxford University Press; 1989: 403–407.

[30] Williams EN, Morrow SL. Achieving trustworthiness in qualitative research: A pan-paradigmatic perspective. Psychother Res. 2009; 19(4–5): 576–582. DOI: 10.1080/10503300802702113 19579089

[31] Erdman JN. Human rights education in patient care. Public Health Rev. 2017; 38: 14 DOI: 10.1186/s40985-017-0061-8 29450086 PMC5809882

[32] Cohen J, Ezer T. Human rights in patient care: A theoretical and practical framework. Health Hum Rights. 2013; 15(2): 7–19. 24421170

[33] Ruchman SG, Green A, Schonholz S, et al. A Toolkit for Building Medical Programs for Asylum Seekers: Resources from the Mount Sinai Human Rights Program. Manuscript submitted for publication 2 24, 2020 DOI: 10.1016/j.jflm.2020.102037 32932168

[34] Barrington AJ, Shakespeare-Finch J. Working with refugee survivors of torture and trauma: An opportunity for vicarious post-traumatic growth. Couns Psychol Q. 2013; 26(1): 89–105. DOI: 10.1080/09515070.2012.727553

[35] Hyatt-Burkhart D. The experience of vicarious posttraumatic growth in mental health workers. J Loss Trauma. 2014; 19(5): 452–461. DOI: 10.1080/15325024.2013.797268

